# Supplementary material for: The effect of travoprost on primary human limbal epithelial cells and the siRNA-based aniridia limbal epithelial cell model, in vitro
Source: PLoS One. 2025 Aug 19;20(8):e0330492. doi: 10.1371/journal.pone.0330492 (PMC12364336; doi:10.1371/journal.pone.0330492)
Supplement: Attachment — Submitted filename: Response to Reviewers.docx [file pone.0330492.s002.docx]

Homburg, 19^th^ July 2025

Dear Prof. Dr. Alexander V Ljubimov,

Thank you so much for reviewing our manuscript, entitled **“The Effect of Travoprost on Primary Human Limbal Epithelial Cells and the siRNA-based Aniridia Limbal Epithelial Cell Model, *In Vitro*”** (PONE-D-25-21972).

We have attached our detailed answers to the Editor and Reviewers’ comments.

We hope that, with our changes in the manuscript (marked in green), our work will be suitable for publication in “PLOS ONE”.

We are looking forward to your response; thank you again, for your efforts.

Sincerely yours,

Shuailin Li

(On behalf of all co-authors)

E-mail: shuailinli97@gmail.com

Dr. Rolf M. Schwiete Center for Limbal Stem Cell and Congenital Aniridia Research, Saarland University, Kirrberger Str. 100, Homburg, Saar, 66424, Germany

Phone: +49-6841-1621217

Fax: +49-6841-1612310

*Reviewer #1:*

1. *Please justify the 20 min timepoint selected for travoprost treatment. This seems to be a very short exposure of the drug and does not accurately mimic clinical settings.*

We thank the reviewer for this insightful comment. In vivo studies in rabbits have shown that plasma concentrations of travoprost peak within 10 to 30 minutes after administration and then decline rapidly (Waugh J, et al. *Drugs Aging.* 2002;19(6):465–473). In our preliminary experiments, we treated limbal epithelial cells (LECs) with various concentrations of travoprost for 10, 20, and 30 minutes, respectively. These tests revealed that a 10-minute exposure had no discernible effect on LEC viability, whereas a 30-minute exposure resulted in a marked reduction in cell viability, which in turn made it challenging to collect sufficient cells for subsequent Western blot and qPCR analyses.

Taking these observations into account, we selected a 20-minute exposure as a representative short-term in vitro treatment model. This duration aligns with several previous in vitro studies that investigated the effects of prostaglandin analogs on corneal epithelial cells (Paimela T, et al. *Mol Vis.* 2012;18:1189–1196; Whitson JT, et al. *Adv Ther.* 2012;29(10):874–888). Additionally, one of our prior studies employed a similar 20-minute travoprost treatment to examine effects on limbal stromal cells (Li S, et al. *PLoS One.* 2025;20(6):e0326967).

Nonetheless, we fully agree that studies involving chronic exposure are essential to more accurately reflect clinical conditions. We have now acknowledged this limitation in the Discussion section and highlighted the need for future in vivo and long-term exposure studies.

2. *The authors should discuss why cell viability is decreased even at lower concentrations.*

We appreciate the reviewer’s suggestion. We have incorporated the required information into the Discussion section, as follows:

“At lower concentrations, the reduction in LEC viability induced by travoprost may not result from overt cytotoxicity but rather from the early activation of stress- and apoptosis-related signaling pathways. In PAX6-knockdown LECs, we observed an increase in caspase-3 protein levels following treatment with 0.313 μg/mL travoprost, supporting the hypothesis that travoprost can trigger apoptotic processes even at relatively low concentrations. This early response appears to be more pronounced in PAX6-deficient cells, which exhibit impaired regulatory capacity and heightened sensitivity to external stimuli. Similarly, we found that low-concentration travoprost treatment elevated JNK protein levels in PAX6-knockdown LECs, suggesting that travoprost may also induce mild oxidative stress under these conditions [37].”

3. *The results section needs to be re-written for clarity. The performed experiments need a rationale.*

Thank you for this valuable feedback. We have restructured the Results section to enhance clarity by introducing a clear rationale at the beginning of each subsection. These revisions are intended to guide the reader more effectively through the experimental design and the interpretation of our findings.

4. *Figure 3 images have low resolution. Please increase the resolution when making the tiff file.*

Thank you very much. We have replaced Figure 3 with higher-resolution TIFF images to meet publication standards, and these updated images are included in the revised submission.

5. *Please clarify whether travoprost affects cell proliferation in siPAX6 LEC, not just cell migration.*

Thank you for pointing this out. Performing PAX6 knockdown in 96-well plates presents certain technical challenges, which is why we did not assess the effect of travoprost on the proliferative capacity of PAX6-knockdown LECs in this study.

However, we have now included a comparative analysis of Ki67 mRNA levels following travoprost treatment in the revised Results section (**Figure 5**). While the primary suppression of proliferation was observed in normal LECs at higher travoprost concentrations, no statistically significant changes in Ki67 mRNA levels were detected in either LECs or PAX6-knockdown LECs treated with 0.156 or 0.313 μg/mL travoprost. This clarification has been incorporated into both the Results and Discussion sections.

**“3.4 *Ki67* mRNA levels**

Ki67 is widely regarded as a marker of cell proliferation [32]. The mRNA levels of *Ki67* did not differ significantly between LECs and PAX6-knockdown LECs (p = 0.321). Moreover, treatment with travoprost did not affect *Ki67* mRNA expression in either the LEC or PAX6-knockdown subgroups (p ≥ 0.867) (Fig 5).

**Fig 5. *Ki67* mRNA levels in limbal epithelial cells (LECs) and PAX6-knockdown LECs following a 20-minute treatment with travoprost at concentrations of 0.156 μg/mL and 0.313 μg/mL (n=7).** mRNA values are presented on a logarithmic scale (Log₂) and expressed as the geometric mean±geometric standard deviation (SD). Two-way ANOVA was used for statistical analysis, and significant p-values (p<0.05) are indicated in the diagrams.

No significant differences were observed between any of the analyzed groups or subgroups (p≥0.321).”

“We also examined the effects of 0.156 and 0.313 μg/mL travoprost on Ki67 mRNA levels, a marker of cell proliferation [32], in both LECs and PAX6-knockdown LECs. These travoprost concentrations did not affect proliferation in either group, consistent with our BrdU assay results.”

6. *Please revisit the Figure 4, 0.313 ug/mL travoprost treatment: resolve the discrepancy between an increase in PAX6 expression and the bar graph.*

We thank the reviewer for bringing this to our attention. We found no significant increase in PAX6 expression after treatment with 0.313 μg/mL travoprost in either LECs or PAX6-knockdown LECs. **Figure 4** now includes a representative Western blot image for clarity.

7. *Please provide rationale for measuring the expression of several genes/proteins like FOSL1, ERK1, ERK2, JNK, and MMP-9 that have been shown without any prior mention.*

Thank you very much. We appreciate this suggestion and have now revised the Introduction to more clearly justify our focus on these targets:

“Fos like 2 (FOSL2) is considered a target gene of PAX6 and is closely associated with corneal opacity [21]. It is also thought to play a role in regulating cell migration and proliferation [22]. Mitogen-activated protein kinases (MAPKs) are primarily categorized into three subfamilies: extracellular signal-regulated kinases (ERKs), c-Jun N-terminal kinases (JNKs), and mitogen-activated protein kinase 14 (MAPK14, also known as p38) [23]. Among these, ERKs and JNKs are particularly implicated in controlling cell proliferation, migration, and apoptosis [24–26]. As previously noted, travoprost can elevate MMP levels, thereby contributing to the reduction of IOP [27]. Related studies have also demonstrated that MMP expression is regulated through the JNK pathway [28]. In our earlier investigation of aniridia limbal stromal cells (AN-LSCs), we found that travoprost may influence MMP9 expression via the JNK signaling pathway, subsequently affecting cell migration [29]. Based on these findings, the present study focuses on evaluating the expression of these key genes and proteins.”

8. *In Figures 5 and 6, please comment on why the expression of several markers are changed at the mRNA level but not at the protein level. This could be because the authors have measured the expression of total proteins (e.g., ERK and NF-kB) but not the phosphorylated (activated) forms.*

Thank you for this important observation. The reviewer correctly noted the discrepancies where certain markers showed differences at the mRNA level but not at the protein level. We acknowledge that for some proteins, such as ERK and NF-κB, we only measured total protein levels and did not assess their phosphorylated (active) forms. Given that we observed no differences between the subgroups treated with varying concentrations of travoprost, we did not extend our analyses in the present study to include phosphorylated protein levels. Nonetheless, we have now added a note in the Discussion section acknowledging this as a limitation of our study, emphasizing that the active (phosphorylated) forms of ERK and NF-κB were not assessed and warrant further investigation:

“In the present study, the limited amount of available material prevented us from measuring the active, phosphorylated forms of ERK and NF-κB, highlighting the need for further investigation.”

9. *There are several pathways that mediate cell proliferation and migration (e.g., Akt or Wnt). The authors have not explained why ERK was selected over other pathways. Please check some other effectors.*

We thank the reviewer for this valuable suggestion. Our initial focus on ERK and JNK was based on their well-established roles in ocular epithelial wound healing and their reported regulation by prostaglandin analogs. We agree that examining additional pathways would provide a more comprehensive understanding. Nevertheless, working with primary cells provided only limited material, which made it unfeasible to analyze all pathways in this study. Accordingly, we have added a note in the Discussion acknowledging as a limitation of our current study that neither the Akt nor Wnt pathways were analyzed, and we propose investigating these pathways in future research:

“For the same reason, we were also unable to analyze the Akt and Wnt pathways, which we propose to investigate in future studies.”

10. *The Quantitative PCR should be Quantitative RT-PCR.*

Thank you for pointing this out. We have corrected all instances of "Quantitative PCR" to "Quantitative RT-PCR" throughout the manuscript.
